# Bedside Ultrasound in Workup of Self-Inserted Headset Cable into the Penile Urethra and Incidentally Discovered Intravesical Foreign Body

**DOI:** 10.1155/2013/587018

**Published:** 2013-12-25

**Authors:** Ali Hajiran, Dana C. Point, Stanley Zaslau

**Affiliations:** Division of Urology, West Virginia University, P.O. Box 9238, Morgantown, WV 26506, USA

## Abstract

There are multiple reports of foreign bodies inserted into the lower urinary tract. We report the case of an incidentally discovered foreign body identified within the bladder in a male patient presenting with a radio antenna protruding from the urethra attached to a head set. On workup patient was found to have an additional foreign body within the bladder and second radiolucent object within the urethra. This case demonstrates the importance of complete evaluation of the lower urinary tract during workup of inserted foreign bodies and the value of the bedside ultrasound as a diagnostic tool in distinguishing between rectal and genitourinary tract insertion.

## 1. Introduction

Self-insertion of foreign objects in the lower genitourinary tract is a rare but well-documented occurrence in the urologic literature. There have been multiple cases of sharp objects (hair pins, tweezers, screws, nails, and fish hooks), large objects (AAA batteries, garden hoses, toothbrushes, and ballpoint pens), and organic materials (carrots, cucumbers, bamboo sticks, and leaves) discovered in the urethra and bladder of patients presenting to the emergency department [1–7]. While some patients may provide an accurate history and exhibit visible pathology on examination, many patients will present with nonspecific symptoms and provide poor histories due to either embarrassment or limited mental capacity [4, 8–10]. Subsequently, a high index of suspicion must be maintained in order to properly diagnose and manage patients with self-inserted foreign objects. We present a case of a developmentally delayed 64-year-old male with a month-long history of recurrent urinary tract infections. He presented to the emergency department with fishing line and a black electrical cable inserted into his penile urethra with an intact headset attached externally. Prompt bedside ultrasound revealed a second radioopaque object in the bladder. An anesthetic penile block was performed to facilitate removal of the fishing line and cable with gentle traction, followed by a bedside cystoscopy to retrieve a separate intravesicular coil of copper wire. All foreign bodies were safely removed without complications. The patient was subsequently discharged with a five-day course of prophylactic antibiotics. We suggest implementing bedside ultrasound as a quick, low-cost, and effective initial screening tool to evaluate all patients presenting with urethral foreign bodies to help rule out the possibility of additional objects in the urinary bladder.

## 2. Case Report

**Figure 1:**
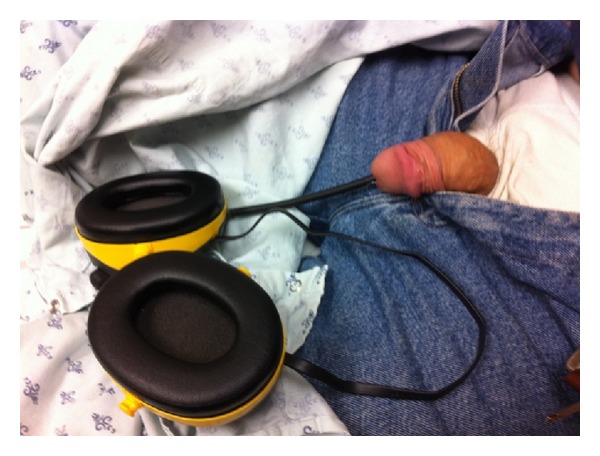
Patient on initial presentation with headset antenna inserted into the penile urethra. A piece of transparent fishing line was also found to be protruding from the urethral meatus alongside the atenna; however, it is not visible in this figure.

**Figure 2:**
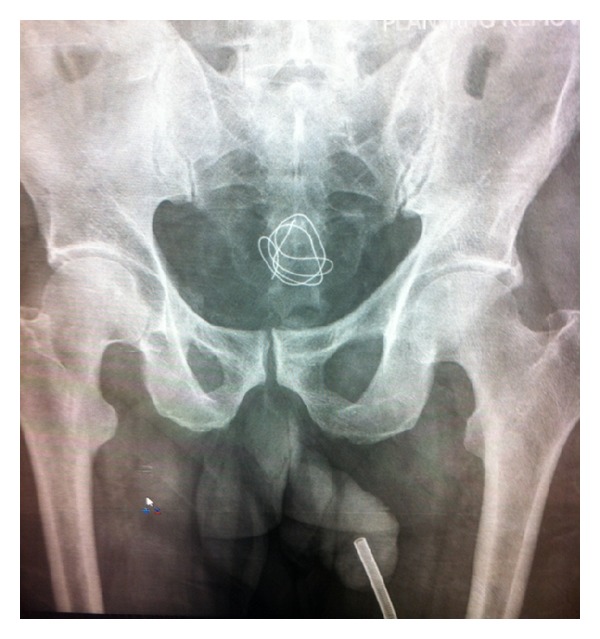
Plain film demonstrating antenna in penile urethra and radiopaque foreign body within the pelvis. Of note the fishing line with attached beads was not visible.

**Figure 3:**
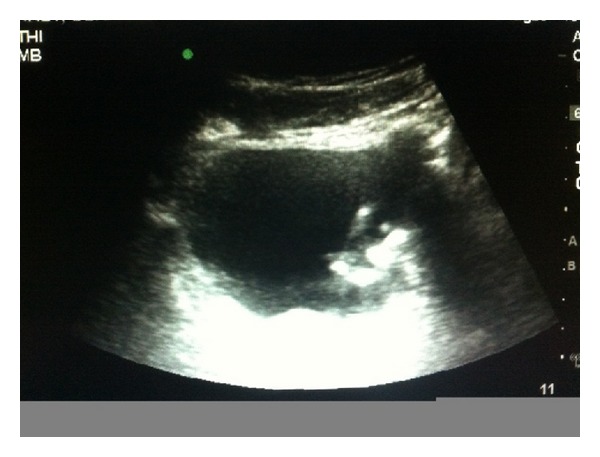
Bedside ultrasound demonstrating hyperechoic object within the bladder.

**Figure 4:**
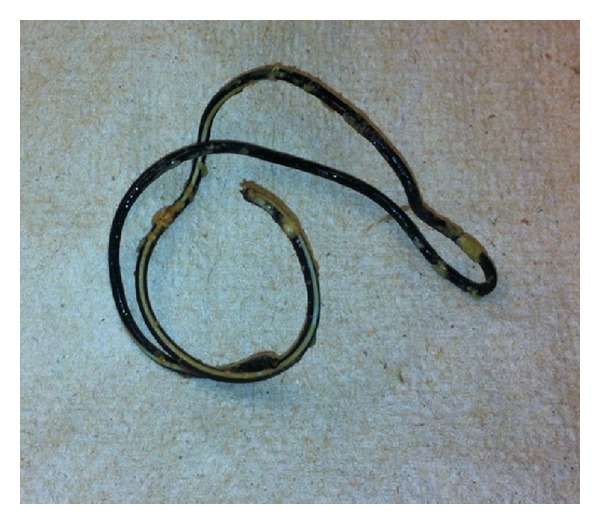
Electric wire with calcifications removed by bedside cystoscopy.

A developmentally delayed 64-year-old gentleman with a history of recurrent urinary tract infections presented to the emergency department with an intraurethral foreign body. According to the patient's caregiver, he had been experiencing intermittent symptoms of suprapubic pain, dysuria, and low-grade fevers for the past six weeks. At an outside facility, he was diagnosed with recurrent urinary tract infections and was treated with empiric antimicrobial therapy without improvement. On the morning of presentation, the patient was found by his caregiver to have a fishing line and a black electric cable protruding from his penile urethra with an intact headset attached externally (Figure 1). The patient was known to have a hobby of making necklaces out of fishing line and beads. He lived in a group home and also had a history of inserting foreign objects in his rectum but had no known history of inserting objects in his urinary tract. Upon questioning, the patient could not articulate his motivation for inserting these objects into his lower urinary tract. On examination the antenna was palpable to the distal half of the penis and a plain pelvic X-ray demonstrated that the antenna did not extend beyond the penile urethra. A radioopaque coil was noted within the pelvis (Figure 2). Prompt bedside ultrasound in the emergency department revealed a separate coil of radioopaque material in the bladder (Figure 3). An anesthetic penile block was performed and the fishing line with two plastic beads attached and the antenna of the headset were successfully removed from the penile urethra using gentle traction. Bedside cystoscopy was performed to remove a coil of electrical wire with mild calcifications from the bladder (Figure 4). Following the procedure, the patient was able to void without difficulty. The patient was subsequently discharged home from the emergency department with a five day course of antibiotics.

## 3. Discussion

According to Moon et al. [11], a foreign body must travel approximately 20 to 25 cm to pass from the urethral meatus to the urinary bladder in an adult erect penis. Given the protected position of the bladder along with the curvature of the bulbar urethra, it is striking to see cases of objects deep in the urethra or urinary bladder in males. A review of 1,272 cases found that men are 1.7 times more likely to self-insert foreign bodies into their lower genitourinary tract than women [12]. Sexual curiosity, autoerotic impulses, intoxication, and psychiatric illnesses are the most common underlying motivations reported in the literature [2–5, 9, 11]. Sinopidis et al. [13] suggested that widespread internet access may lead to an increased incidence of self-inserted foreign bodies as they present a case of a 12-year-old male who inserted an electrical television wire into his urethra after reading an online paper which falsely claimed that it would augment penile length and provide erotic gratification.

The clinical presentation of a distal intraurethral foreign body may be straightforward with obvious genital swelling and visible material protruding from the urethral meatus on examination. However, the diagnosis of a foreign object in the proximal urethra or urinary bladder can present more of a diagnostic challenge. Nonspecific urologic symptoms and an inaccurate patient history due to embarrassment or limited mental capacity can create a diagnostic dilemma [4, 8–10]. The most common symptoms of an intravesicular foreign body are increased urinary frequency, dysuria, hematuria, suprapubic discomfort, and intermittent low-grade fevers. However, some patients may harbor intravesicular objects for weeks to months, while experiencing minimal to no symptoms [14]. All patients with suspected genitourinary foreign bodies require radiographic imaging to determine the location, size, and number of foreign objects. While initial treatment includes analgesics for pain control and management of urologic symptoms with anticholinergic medications or catheterization, the results of the radiological evaluation will ultimately determine which intervention is required [6, 10]. Today, many foreign objects can be safely removed using minimally invasive endoscopic techniques. Other foreign bodies may require open procedures, such as an external urethrotomy or suprapubic cystotomy, depending on the size and nature of the object [6, 15]. For followup, some authors recommend routine psychiatric evaluation; however, this remains controversial as many patients do not have an underlying psychological illnesses [10, 15–19].

Our patient presented to the emergency department with a visible intraurethral foreign body. Plain films served as a good initial evaluation; however, bedside ultrasound was able to confirm the second radioopaque objects location within the urinary bladder. The intravesicular coil of electrical wire was likely the culprit of the patient's recurrent urinary tract infections and ongoing urologic symptoms for the previous month. There have been other reported cases of men inserting multiple foreign bodies in the urethra and urinary bladder [11]. Failure to detect additional objects in a patient presenting with an intraurethral foreign body may lead to delayed abscess formation, sepsis, perforation, or death [20]. We recommend implementing bedside ultrasound as a quick, low-cost, and effective initial screening tool to evaluate all patients presenting with urethral foreign bodies to help rule out the possibility of additional objects in the urinary bladder.

## 4. Conclusions

Lower genitourinary foreign bodies represent an unusual but well-documented form of pathology in the field of urology. Failure to detect and remove all objects from a patient's urethra and bladder can lead to chronic or fatal complications. A complete history, examination, and imaging to completely evaluate the lower urinary tract are key to making a complete diagnosis. Plain films are limited in that they will not show radiolucent foreign bodies and may not differentiate between rectal and genitourinary pathology. We recommend bedside bladder ultrasound as a cost effective imaging tool in the workup of this unusual phenomenon.
